# Supplementary material for: Restoration of T and B Cell Differentiation after RAG1 Gene Transfer in Human RAG1 Defective Hematopoietic Stem Cells
Source: Biomedicines. 2024 Jul 5;12(7):1495. doi: 10.3390/biomedicines12071495 (PMC11275127; doi:10.3390/biomedicines12071495)
Supplement: Supplementary file 1 [file biomedicines-12-01495-s001.zip › biomedicines-2986534-supplementary.pdf]

Figure S1. Test of the RAG1 GMP lentiviral batch on healthy donor (HD) samples.

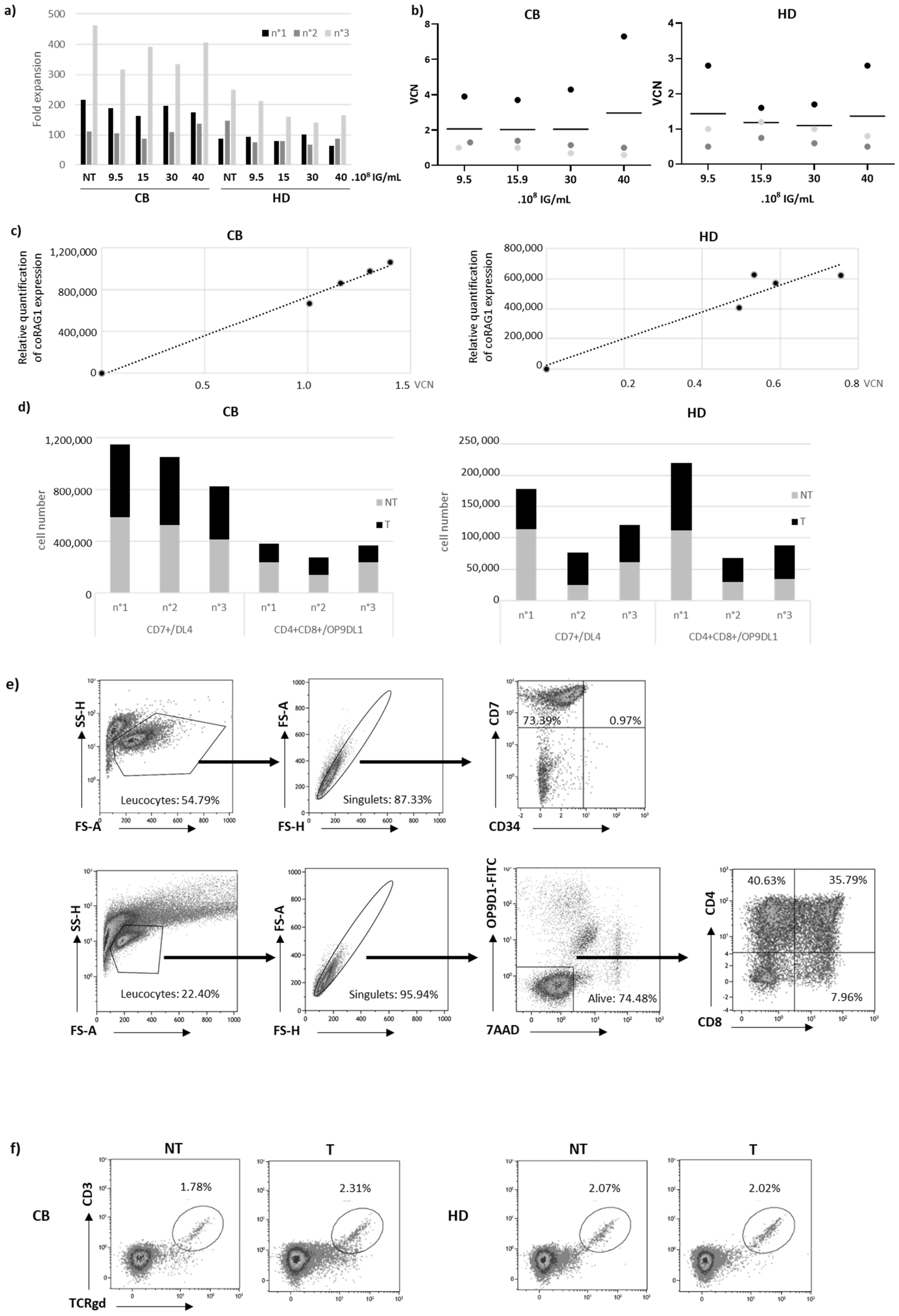

Figure S2. Immune reconstitution in NSG mice with healthy donor (HD) samples (16 weeks post injection).

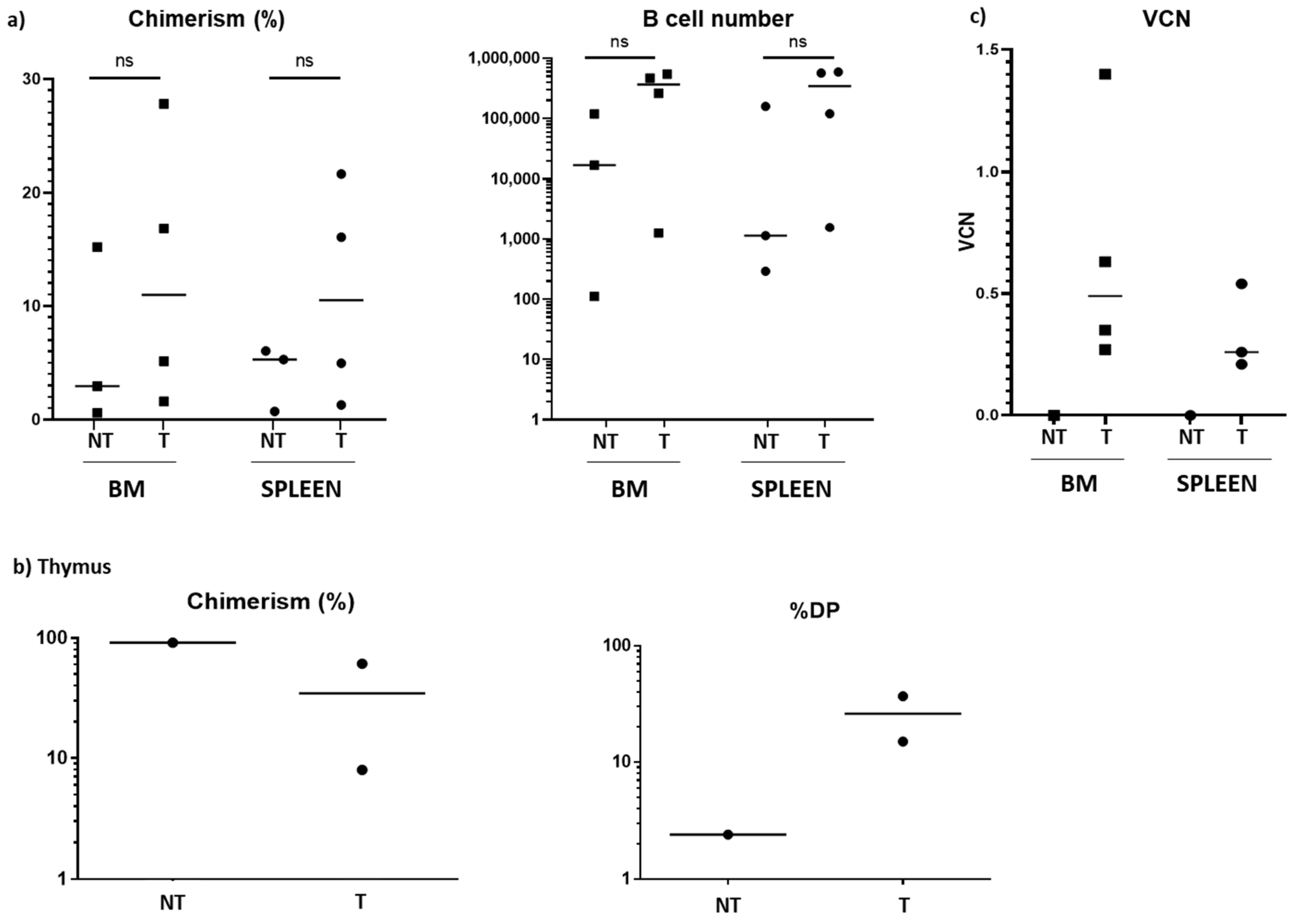

Figure S3. Immune reconstitution in NSG mice (16 weeks)-P1.

a) TCR repertoire diversity distribution in the spleen

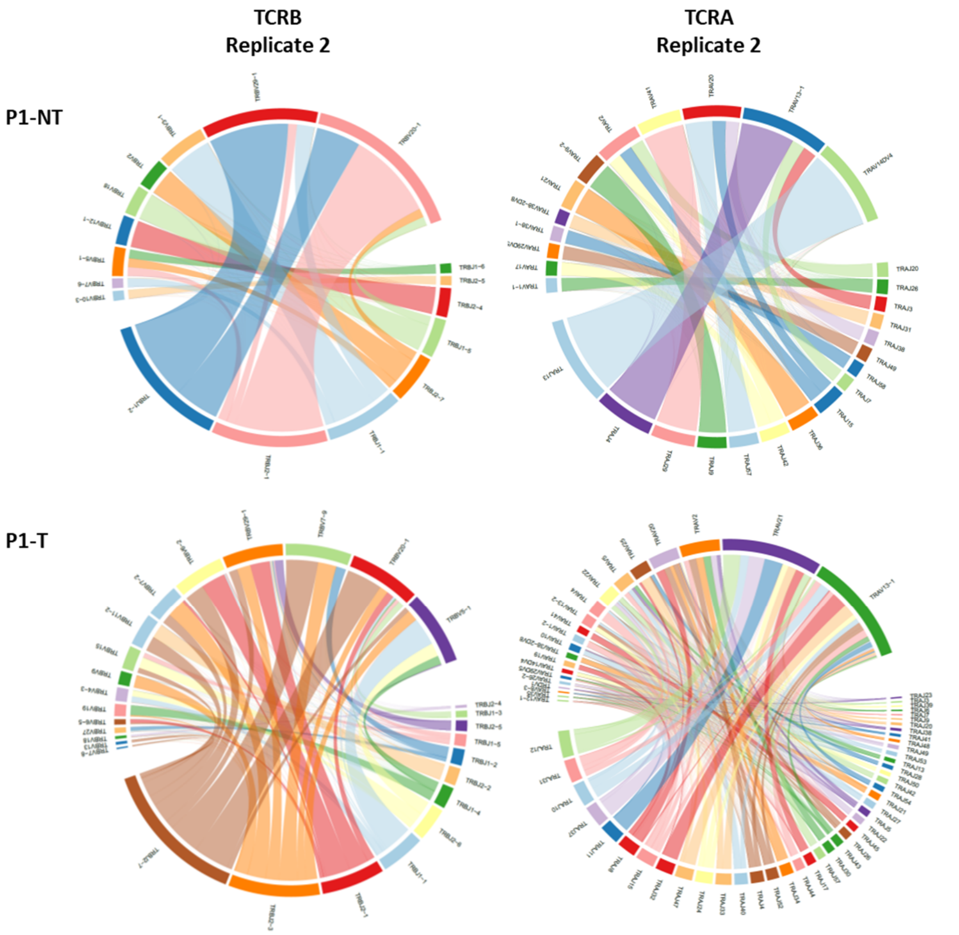

b) CD3R length distribution in the spleen

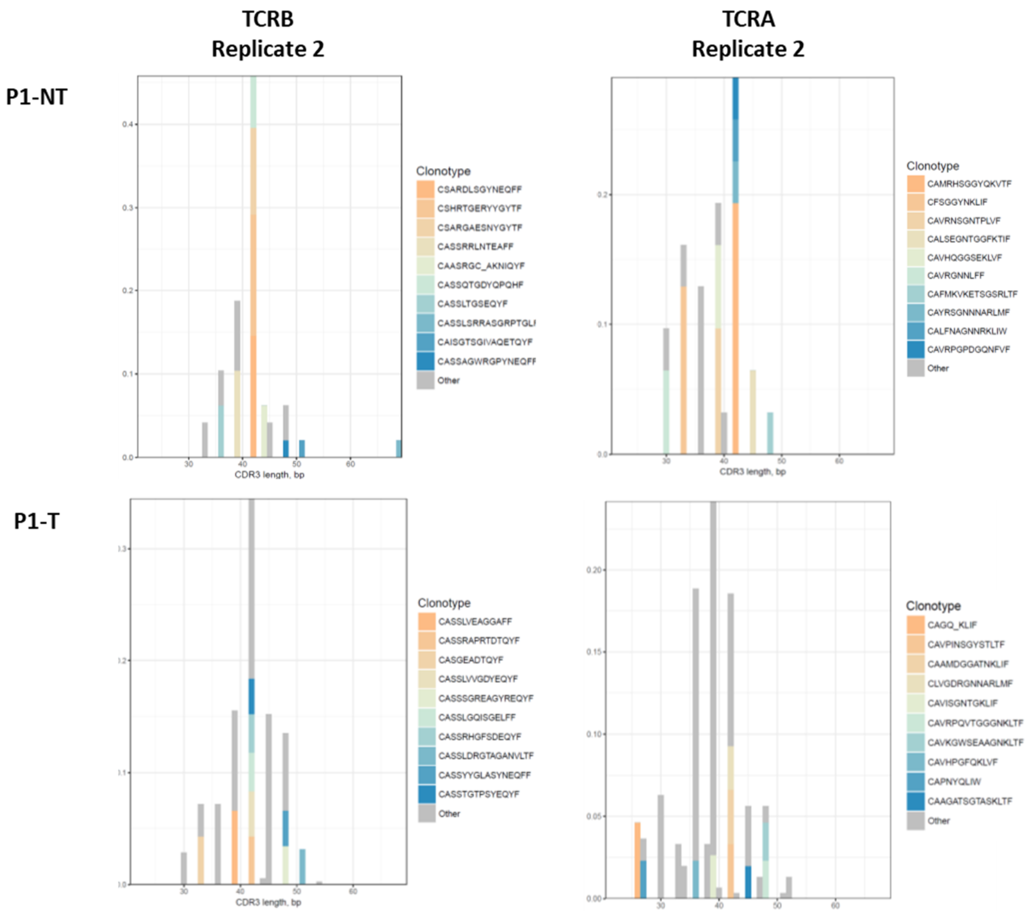

Table S1 : Common integration sites (CIS) in the RAG1 transduced cells from NSG mice injected with P1 or P2 cells and in the CD34+ of P2 after a 14-days *in vitro* culture

**Top10 CIS P1**	CIS Order	Chromosome	Average Position	Dimension [nt]	Gene
Top 1	4	19	15,382,832	47	*AKAP8L*
Top 2	3	12	49,422,490	60	*SPATS2*
Top 3	3	22	50,557,324	153	*SYCE3*
Top 4	3	11	65,515,808	24,843	*MALAT1, SCYL1*
Top 5	2	11	350,704	0	*B4GALNT4*
Top 6	2	12	29,184,977	0	*FAR2*
Top 7	2	16	29,661,222	0	*SPN*
Top 8	2	17	31,202,767	0	*NF1*
Top 9	2	17	37,902,318	0	*YWHAEP7*
Top 10	2	19	48,235,459	0	*CARD8*

**Top10 CIS P2**	CIS Order	Chromosome	Average Position	Dimension [nt]	Gene
Top 1	3	1	29,016,369	103	*EPB41*
Top 2	3	7	158,888,062	146	*WDR60*
Top 3	2	20	9,105,365	14,200	*PLCB4*
Top 4	2	17	7,240,821	15,704	*CTDNEP1, DVL2*
Top 5	2	1	97,724,680	15,774	*DPYD*
Top 6	2	19	14,010,910	18,514	*RFX1, RLN3*
Top 7	2	16	1,727,365	19,059	*MAPK8IP3*
Top 8	2	17	41,156,793	28,969	*KRTAP4-3, KRTAP9-7*
Top 9	2	4	173,444,262	37,393	*SCRG1*
Top 10	2	19	1,180,394	40,953	*SBNO2, STK11*

**Top10 CIS P2 CD34+**	CIS Order	Chromosome	Average position	Dimension [nt]	Gene
Top 1	6	17	78,112,859	86,795	*C17orf99, TNRC6C*
Top 2	5	19	49,618,818	31,843	*PRR12, RRAS*
Top 3	5	16	237,358	36,557	*ITFG3, LUC7L*
Top 4	4	16	53,199,234	53,577	*CHD9*
Top 5	4	16	88,708,489	81,797	*PIEZO1, RNF166, SNAI3-AS1*
Top 6	3	17	75,791,130	3666	*UNK*
Top 7	3	23	79,165,630	4352	*GPR174*
Top 8	3	7	87,842,938	8417	*SLC25A40*
Top 9	3	17	12,105,848	8902	*MAP2K4*
Top 10	3	16	2,252,522	16,344	*ECI1, RNPS1*

Table S2 : Cancer genes in the RAG1 transduced cells from NSG mice injected with P1 or P2 cells and in the CD34+ of P2 after a 14-day *in vitro* culture

Cancer Gene IS P1	Sample	Cancer Gene	Frequency [%]	Sample	Cancer Gene	Frequency [%]
Top 1	T BM	*BRD4*	26.033	T Spleen	*BRD4*	24.190
Top 2		*NF1*	4.623		*NF1*	6.824
Top 3		*MALAT1*	2.106		*MALAT1*	0.555
Top 4		*BRCA1*	0.407		*MALAT1*	0.027
Top 5		*ETV4*	0.151		*MAP2K2*	0.014
Top 6		*NOTCH1*	0.005		*MAPK1*	0.007
Top 7		*TCF12*	0.004		*AKT2*	0.002
Top 8		*BRD4*	0.001		*BRD4*	0.001
Top 9		N/A	N/A		*STK11*	0.001
Top 10					N/A	N/A

Cancer Gene IS P2	Sample	Cancer Gene	Frequency [%]	Sample	Cancer Gene	Frequency [%]
Top 1	T4 BM	*STK11*	5.188	T4 Spleen	*BIRC6*	26.905
Top 2		*ARNT*	0.994		*STAT3*	8.383
Top 3		*SETDB1*	0.994		*STAT3*	8.383
Top 4		*ETNK1*	0.908		*PRKCB*	3.476
Top 5		*PRKCB*	0.760		*RFX1*	2.750
Top 6		*C15orf65*	0.540		*INHBC*	0.017
Top 7		*PSIP1*	0.382		*INHBC*	0.017
Top 8		*NT5C2*	0.329		*TULP2*	0.007
Top 9		*ERG*	0.325		N/A	N/A
Top 10		*MEN1*	0.319		N/A	N/A

Cancer Gene IS P2	Sample	Cancer Gene	Frequency [%]	Sample	Cancer Gene	Frequency [%]
Top 1	T6 BM	*AKT2*	9.210	T6 Spleen	N/A	N/A
Top 2		*CIC*	4.622		N/A	N/A
Top 3		*FSTL3*	2.758		N/A	N/A
Top 4		*EPS15*	1.902		N/A	N/A
Top 5		*BRAF*	1.004		N/A	N/A
Top 6		*BLM*	0.155		N/A	N/A
Top 7		*DDX3X*	0.125		N/A	N/A
Top 8		*TCF12*	0.123		N/A	N/A
Top 9		*PRKACA*	0.007		N/A	N/A
Top 10		*RUNX1*	0.001		N/A	N/A

**Cancer Gene IS P2**	Sample	Cancer Gene	Frequency [%]	Sample	Cancer Gene	Frequency [%]
Top 1	T7 BM	*ZNF331*	4.127	T7 Spleen	*PMS2*	6.164
Top 2		*AKT2*	0.367		*MUTYH*	1.302
Top 3		*PRPF40B*	0.363		*ZNF331*	0.024
Top 4		*MALAT1*	0.103		*MYD88*	0.001
Top 5		*KTN1*	0.084		N/A	N/A
Top 6		*FAT4*	0.062		N/A	N/A
Top 7		*ZNF331*	0.016		N/A	N/A
Top 8		*PDGFB*	0.002		N/A	N/A
Top 9		*PMS2*	0.001		N/A	N/A
Top 10		N/A	N/A		N/A	N/A

T4, T6 and T7 represent the 3 mice that received CD34+ transduced cells from P2.

**Cancer Gene IS P2 HSPCs**	Cancer Gene	Frequency [%]
Top 1	*DDX5*	0.366
Top 2	*CBLB*	0.275
Top 3	*ERC1*	0.275
Top 4	*RHOH*	0.275
Top 5	*KDM6A*	0.275
Top 6	*FAS*	0.275
Top 7	*N4BP2*	0.275
Top 8	*BIRC6*	0.183
Top 9	*RAD51B*	0.183
Top 10	*MAP2K4*	0.183
